# Characterization of CobB kinetics and inhibition by nicotinamide

**DOI:** 10.1371/journal.pone.0189689

**Published:** 2017-12-18

**Authors:** Julia Gallego-Jara, Ana Écija Conesa, Teresa de Diego Puente, Gema Lozano Terol, Manuel Cánovas Díaz

**Affiliations:** Department of Biochemistry and Molecular Biology and Immunology (B), Faculty of Chemistry, University of Murcia, Campus of Espinardo, Regional Campus of International Excellence ‘‘Campus Mare Nostrum”, Murcia, Spain; Saint Louis University School of Medicine, UNITED STATES

## Abstract

Lysine acetylation has emerged as a global protein regulation system in all domains of life. Sirtuins, or Sir2-like enzymes, are a family of histone deacetylases characterized by their employing NAD^+^ as a co-substrate. Sirtuins can deacetylate several acetylated proteins, but a consensus substrate recognition sequence has not yet been established. Product inhibition of many eukaryotic sirtuins by nicotinamide and its analogues has been studied *in vitro* due to their potential role as anticancer agents. In this work, the kinetics of CobB, the main *Escherichia coli* deacetylase, have been characterized. To our knowledge, this is the first kinetic characterization of a sirtuin employing a fully acetylated and natively folded protein as a substrate. CobB deacetylated several acetyl-CoA synthetase acetylated lysines with a single kinetic rate. In addition, *in vitro* nicotinamide inhibition of CobB has been characterized, and the intracellular nicotinamide concentrations have been determined under different growth conditions. The results suggest that nicotinamide can act as a CobB regulator *in vivo*. A nicotinamidase deletion strain was thus phenotypically characterized, and it behaved similarly to the Δ*cobB* strain. The results of this work demonstrate the potential regulatory role of the nicotinamide metabolite *in vivo*.

## Introduction

Protein lysine acetylation is a post-translational modification (PTM) in which the scientific community is increasingly interested [1–3]. Lysine acetylation consists of the transfer of an acetyl group from a donor molecule such as acetyl-CoA or acetyl-phosphate to a lysine amino acid of a protein. This transfer can be catalysed by an acetyltransferase enzyme (KAT) [4,5] or occur in a non-enzymatic or chemical manner [6,7]. The number of proteins that are modified post-translationally by acetylation is continuously discovered in all types of organisms. This PTM has traditionally been associated with transcriptional regulation (histone modifications) [8], although in the past 10 years, many metabolic routes have been demonstrated to be regulated by lysine acetylation [9–15].

Protein acetylation can be reversed by a family of enzymes known as the lysine deacetylases (KDACs). KDACs can be classified into two main groups. The first group acts in a Zn^2+^-dependent manner, while the members of the second group, also known as sirtuins or Sir2-like enzymes, deacetylate lysines in a reaction that employs oxidized nicotinamide adenine dinucleotide (NAD^+^) [16,17] as a co-substrate. Sirtuins remove an acetyl group from the ɛ-amino group of a lysine in a reaction that consumes NAD^+^ and releases nicotinamide (NAM), *O*-acetyl-ADP-ribose, and the deacetylated protein [18]. Many eukaryotic species possess multiple sirtuin paralogs (human sirt1-7) that differ in structure and cell localization. Archaea and bacteria also produce sirtuins, e.g., the bacterial CobB [19–21]. In *Escherichia coli* (*E*. *coli*), the most studied deacetylase is CobB, a bacterial sirtuin that was first characterized in *Salmonella enterica* [21]. Other *E*. *coli* proteins such as acetyl-CoA synthetase (Acs) and phosphotransacetylase (Pta) have been reported to functionally act as deacetylases of the chemotaxis signalling molecule CheY, although, to our knowledge, there is not a proteomic study that shows a deacetylase activity with other substrates [22,23]. In addition, it has recently been demonstrated that YcgC deacetylates several acetylated lysines from different proteins without needing NAD^+^ or Zn^2+^ as co-substrates [24]. Sirtuins share a common structural core formed by a Rossman domain, a Zn^2+^-finger domain and a connector loop. The N- and C-terminal regions differ in sequence and length, which might determine differences in substrate specificity [25].

With respect to sirtuin substrates, eukaryotic sirtuins have been linked to cell survival, apoptosis, and stress resistance [26,27]. The typical substrates of archaea and bacteria sirtuins are DNA regulatory proteins (such as the chromatin protein Alba) and metabolic enzymes (such as acyl-CoA synthetases) [2,21,28–33]. In *E*. *coli*, Acs regulation by acetylation-deacetylation has been studied *in vitro* [15,34,35]. Thus, Acs is acetylated at K609 by the acetyltransferase YfiQ/Pka/PatZ inhibiting its catalytic activity, while enzymatic activity is recovered by CobB deacetylation [34–36]. The CheY chemotaxis response regulator [37,38], the Nhoa protein [39] and the RcsB transcription factor [15,40] have also been identified as CobB substrates. Recent proteomic studies have identified as many as 69 acetyl-lysine residues among 51 proteins to be CobB substrates [7] and have also found 183 proteins that interact with this sirtuin [41]. Several studies have tried to identify a consensus recognition sequence for CobB and other sirtuins [18,42–48], but the results are not clear and do not always correspond to proteomic studies performed *in vivo* [7,15,49,50]. Due to the interest in sirtuins as targets for the treatment of metabolic diseases [51,52], several studies have addressed the regulation and inhibition of these enzymes. Nicotinamide, a product of the sirtuin catalytic reaction, is a potent inhibitor for all of these enzymes. Although the mechanism of inhibition by NAM is not completely clear, most of the *in vitro* studies carried out with eukaryotic sirtuins point to non-competitive inhibition via a base-exchange mechanism that involves reacting with an intermediate to reform NAD^+^ (transglycosidation mechanism) [53–56].

Herein we carry out a CobB sirtuin study by kinetically characterizing acetylated-Acs deacetylation. To our knowledge, this report is the first kinetic characterization of a sirtuin deacetylation reaction with a complete natively folded substrate. In addition, we studied CobB inhibition by nicotinamide *in vitro* and *in vivo*. The results of this work represent an advance in the study of sirtuin specificity and substrate recognition; in addition, our data suggest a new level of CobB regulation *in vivo* via inhibition by nicotinamide, a poorly studied metabolite with a central metabolic role.

## Material and methods

### Molecular biology

To generate overexpression plasmids, the *cobB* and nicotinamidase (*pncA*) genes of *E*. *coli* BW25113 were PCR-amplified and cloned into pBAD24-MBP (Maltose Binding Protein) (kindly transferred by Dr. Antonio Sánchez-Amat, University of Murcia) and pRSETA (Invitrogen) plasmids, respectively. To overexpress Acs protein, the correspondent ASKA plasmid was employed [57]. Single amino acid mutant Acs K609A was obtained by site-directed mutagenesis from *acs*ASKA plasmid. To carry out the complementation assays, the *pncA* and *cobB* genes were PCR-amplified from *E*. *coli* BW25113 genomic DNA and cloned into the pBAD24 plasmid [58]. All molecular biology enzymes that were used were purchased from Thermo Fisher Scientific. The strains, plasmids and primers used are listed in S1 Table.

### *Escherichia coli* strains and culture conditions

*Escherichia coli* BW25113 and its deletion strains (S1 Table) were grown in batch mode in minimal M9 medium or in complex TB7 medium [59] (10 g/l tryptone buffered at pH 7.0 with 100 mM potassium phosphate) supplemented with glucose (20 mM) or glycerol (40 mM) as the carbon source. Cell growth was monitored spectrophotometrically by determining the optical density at 600 nm (OD_600_). Kanamycin (50 mg/ml), ampicillin (100 mg/ml), and chloramphenicol (30 mg/ml) were added when needed.

### Overexpression and purification of proteins

Chemically competent *E*. *coli* BL21 (DE3) (CobB and nicotinamidase overexpression) or *E*. *coli* BL21 (DE3) Δ*cobB* (Acs and Acs K609A overexpression) strains were transformed by heat shock at 42°C with overexpression plasmids. Cultures were grown overnight at 30°C with orbital shaking (200 rpm). The culture medium that was used was Luria-Bertani broth (LB). Expression was induced with 1 mM isopropyl-β-D-thiogalactopyranoside (IPTG) (*cobB*pBAD24-MBP and *pncA*pRSETA) or 0.1 mM IPTG (*acs*ASKA) when the culture OD_600_ reached 0.5–0.6. Cells were harvested by centrifugation, thoroughly washed with 0.9% NaCl and resuspended in binding buffer (50 mM potassium phosphate, pH 7.5, containing 500 mM NaCl and 20 mM imidazole for IMAC purification and 20 mM Tris-HCl, pH 7.5, containing 200 mM NaCl for amylose purification) that was supplemented with an EDTA-free protease inhibitor (SigmaFast Protease Inhibitor Cocktail Tablet, from Sigma Aldrich). Cells were lysed on ice by sonication for 2 min (20 s each pulse) using a Vibra Cell sonicator (Sonicator Sonics & Materials). The lysates were clarified by centrifugation at 10000 x g for 15 min at 4°C. Recombinant Acs, Acs K609A and nicotinamidase proteins were purified by using immobilized metal affinity chromatography (IMAC). The cell-free extract was loaded onto a 5 ml His GraviTrap column (GE Healthcare), and the column was washed with washing buffer (50 mM potassium phosphate buffer, pH 7.5, containing 500 mM NaCl and 50 mM imidazole). The His_6_-tagged proteins were eluted with an elution buffer (50 mM potassium phosphate buffer, pH 7.5, containing 500 mM NaCl and 500 mM imidazole). CobB sirtuin was purified using an amylose resin (New England Biolabs). The resin was washed with a binding buffer, and CobB was eluted using an amylose elution buffer (20 mM Tris-HCl, pH 7.5, containing 200 mM NaCl and 10 mM maltose). MBP CobB tag was cleaved by incubating the protein with H3V 3C protease (Pierce) over night at 4°C. MBP-tag and protease were removed with a His GraviTrap column. Purified proteins were dialyzed (Pur-a-lyzer dialysis kits) into 50 mM potassium phosphate buffer, pH 7.5, containing 100 mM NaCl and 10% v/v glycerol. Proteins were stored at -80°C until used. Finally, Spin-X UF concentrators (Corning) were employed to concentrate the proteins. Proteins were analysed by using SDS-PAGE electrophoresis with 10% acrylamide gels in a Mini-Protean cell (Bio-Rad).

### Western blot analysis

Proteins with acetylated lysines were separated by using SDS-PAGE and transferred to PVDF membranes using a semi-dry transfer unit (Trans-Blot® SD Semi-Dry Transfer Cell, Bio-Rad). The membranes were incubated with a rabbit monoclonal primary anti-acetyl-lysine antibody (anti-Ac-K) (ImmuneChem) and a goat anti-rabbit IgG secondary antibody (Santa Cruz Biotechnology). Finally, the membrane was incubated for 10 min with Amersham ECL western blotting detection reagent (Thermo Scientific). ImageJ Gel Analyzer software was used for densitometric quantification.

### Liquid chromatography–tandem mass spectrometry assay (LC-MS)

Samples were alkylated by incubation with 100 mM iodoacetamide (IAA) for 30 min at room temperature in the dark. Proteins were digested with 0.5–1 μg of proteomics grade Trypsin Gold (Promega) for 3 h at 37°C. The reaction was stopped by the addition of 0.1% formic acid, and the samples were dried using a vacuum evaporator. Tryptic peptides generated from the samples were separated and analysed by LC-MS. An Agilent 1100 (Agilent Technologies) was equipped with a Zorbax SB-C18 HPLC column (Agilent Technologies) and connected to an Agilent Ion Trap XCT Plus mass spectrometer (Agilent Technologies) that had an electrospray (ESI) interface. Two mobile phases were used: phase A was composed of water/acetonitrile/formic acid (94.9:5:0.1, v/v), and phase B consisted of water/acetonitrile/formic acid (10:89.9:0.1, v/v). The digested peptides were resuspended in 20 μl of phase A and eluted using a linear gradient of 0–80% phase B for 180 min at a flow rate of 10 μl min^-1^. The mass spectrometer was operated in positive mode with a capillary spray voltage of 3500 V, at a scan speed of 8100 (m/z)/sec from 50 to 2200 m/z, with a target mass of 1000 m/z, and three scans were averaged. The nebulizer gas pressure was set at 15 psi, and the drying gas flowed at 5 l min^-1^ at a temperature of 350°C. MS/MS data were collected in an automated data-dependent mode (AutoMS mode). Data processing was performed with the Data Analysis program for LC/MSD Trap Version 3.3 (Bruker Daltonik) and the Spectrum Mill MS Proteomics Workbench (Agilent Technologies) [60,61]. After automated validation of the results, the identified proteins and the sequences of the digested peptides were compiled. Peptides with a score threshold of 8 and a percentage-scored peak intensity higher than 70% were considered valid.

### Acs enzyme assay

The acetyl-CoA synthetase assay was based on the coupled assay reported by Williamson and Corkey [62]. AMP production was detected via a coupled enzyme assay in which myokinase (MK), pyruvate kinase (PK) and lactate dehydrogenase (LDH) couple AMP production to NADH oxidation. Standard acetyl-CoA synthetase assays (0.1 ml) were performed at 37°C in 50 mM potassium phosphate buffer at pH 7.5 and contained 3 mM PEP (phosphoenolpyruvate), 5 units of MK, 1 unit of PK, 1.5 units of LDH, 5 mM MgCl2, 1 mM ATP, 1.5 mM CoA, 0.1 mM NADH, 5 mM acetate and 1 mM dithiothreitol. The reaction was started by the addition of Acs. All reactions were performed in triplicate. Specific activity was calculated using the extinction coefficient of NADH (6.22 mM^-1^ cm^-1^) and was based on the oxidation of two molecules of NADH for each AMP molecule released. One unit of Acs activity is defined as 1 μmole of acetyl-CoA formed per minute at pH 7.5 and 37°C.

### CobB and nicotinamidase enzymatic assay

The continuous CobB assay that was employed was based on the coupled assay published by Denu et al., [63]. In this assay, NAM released by CobB is converted into nicotinamidic acid by nicotinamidase catalysis. A coupled reaction based on glutamate dehydrogenase uses the ammonia and α-ketoglutarate (αKG) to oxidize NADH. Standard CobB assays (0.1 ml) were performed at 37°C in 50 mM potassium phosphate buffer at pH 7.5 and contained 3 mM αKG, 1 mM dithiothreitol, 3 units of GDH (glutamate dehydrogenase), 0.4 mM NADH, 1 μM nicotinamidase and 5 mM NAD^+^. The reaction was started by the addition of CobB. All reactions were performed in triplicate. Specific activity was calculated using the extinction coefficient of NADH (6.22 mM^-1^ cm^-1^) and was based on one molecule of NADH being oxidized for each NAM molecule released. One unit of CobB activity is defined as 1 μmole of NAM formed per minute at pH 7.5 and 37°C.

### Intracellular NAM measurement

Cultures (50 ml) were harvested during the exponential (OD_600_ = 1) or stationary growth phases, and the collected pellets were washed with 0.9% NaCl and stored at -80°C. Nicotinamide extraction was carried out by using a freeze-thaw method with methanol [64]. Cell pellets were resuspended in 1 ml of pure methanol at -20°C with 15 μM d_4_-nicotinamide (deuterated nicotinamide), which was employed as an internal standard. The suspensions were put in liquid nitrogen for 5 min to ensure a completely frozen sample and then on ice for 10 min to thaw them. This freeze-thaw cycle was repeated three times. Then, samples were centrifuged at 16000 rpm and 0°C, and the supernatants were evaporated to dryness. To quantify intracellular metabolites, samples were resuspended in 0.3 ml of HPLC water. The analyses were carried out on an HPLC-MS/MS system consisting of an Agilent 1100 Series HPLC (Agilent Technologies) that was connected to an Agilent Ion Trap XCT Plus mass spectrometer (Agilent Technologies) and used an electrospray (ESI) interface. Samples were injected onto an Agilent Zorbax SB-Aq HPLC column (5 μm, 150 x 4.6 mm) that was thermostatted at 40°C and eluted at a flow rate of 200 μl/min. Mobile phase A, consisting of water with 0.1% formic acid, and mobile phase B, consisting of acetonitrile with 0.1% formic acid, were used for the chromatographic separation. The elution program consisted of 10% phase B for 10 minutes and then a gradient from 10 to 100% of mobile phase B in 15 minutes. Finally, 100% solvent B was maintained for 5 additional minutes. The mass spectrometer was operated in positive polarity mode with a capillary spray voltage of 3500 V and a scan speed of 22000 (m/z)/sec from 50–250 m/z, with the target mass located at 125 m/z. The Smart ICC target was set to 200.000 counts, whereas the maximum accumulation time was 20 msec. The nebulizer gas pressure was set to 30 psi, whereas the drying gas was set to a flow rate of 8 l min^-1^ and the temperature was set to 350°C. The ion chromatograms were extracted, and the peak areas were quantified using the Data Analysis program in LC/MSD Trap Version 3.2 (Bruker Daltonik). The peak area data of the compound in the standards were used to calculate the calibration curve, which was then used to determine the concentrations of NAM and d_4_-nicotinamide in the samples. To determine intracellular NAM concentrations a cell volume of 0.7 μm^3^ was employed and viable cell mass was determined using a linear calibration curve relating optical density at 600 nm and dry cell weight.

### Electron microscopy

*Escherichia coli* strains were grown in TB7-glycerol complex medium. The cells were harvested in mid-exponential phase (OD_600_ = 1) and fixed with 3% glutaraldehyde for 30 minutes. After two washes with 0.9% NaCl, each cell suspension was placed on electron microscopy grids and stained for 15 s with 2% uranyl acetate before their flagella were examined in a Philips Tecnai 12 electron microscope operating at 120 kV.

### Migration assays

Strains were grown TB7-glycerol complex medium until mid-exponential phase (OD_600_ = 1). One microliter of these cultures was inoculated into semisolid agar (10 g/l tryptone, 5 g/l NaCl and 0.25% agar). The migration diameter was measured after 16h at 30°C.

### Extracellular metabolite quantification

Extracellular acetate, glucose and glycerol were analysed by using an HPLC (Shimadzu Scientific Instruments) equipped with differential refractive and UV detectors and a cation-exchange column (HPX- 87H, Bio-Rad). The mobile phase was 5 mM H_2_SO_4_ at 0.5 ml min^-1^ flow rate, and the temperature was 65°C.

## Results

### Kinetics of Acs deacetylation by CobB are monophasic

**Fig 1:**
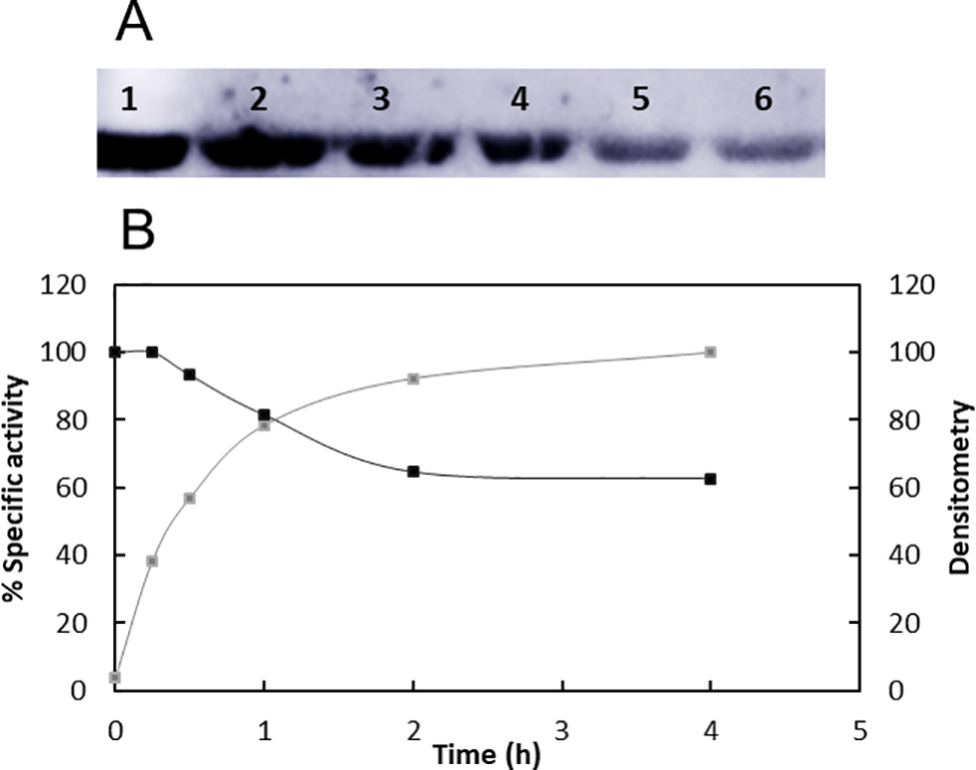
Acs is deacetylated and activated by CobB. **(A)** Western blot with anti-Ac-K of Acs incubated with CobB and NAD^+^ at 0 (lane 1), 0.25 (lane 2), 0.5 (lane 3), 1 (lane 4), 2 (lane 5) and 4 (lane 6) hours of agitation at 37°C. **(B)** Acs specific activity at 0, 0.25, 0.5, 1, 2 and 4 hours (grey curve) and densitometry of Acs western blot (black curve).

Acetyl-CoA synthetase protein was purified from an *E*. *coli* BL21 (DE3) Δ*cobB* strain to achieve a high Acs acetylation level. To ensure that Acs was acetylated, an LC-MS/MS assay was carried out to determine the Acs acetylation state. In total, 15 acetylated lysines were found in the Acs sequence, namely, K68, K111, K130, K200, K207, K221, K226, K348, K370, K400, K555, K585, K604, K609 and K617. These 15 acetylated lysines had been previously detected by other authors [7,15,35,49,65,66]. Acetylated Acs (10 μM) was incubated for 4 h with CobB (800 nM) (12:1 ratio) and NAD^+^ (5 mM). Aliquots were taken and processed for western blot and Acs activity measurements. Acs activity increased about twenty five-fold after 2 hours of incubation with CobB. This increase coincided with a decrease in chemiluminescent signal from the western blot. However, the reaction was not completed to 2 h of incubation. We therefore knew that Acs deacetylation was being partially inhibited, likely by nicotinamide reaction product. The results are shown in Fig 1.

Nicotinamide inhibition of sirtuins is well known and has been studied in many sirtuins [53–56,67,68]. To study the effect of NAM inhibition, *E*. *coli* nicotinamidase or PncA (an enzyme that consumes nicotinamide to generate ammonium and nicotinate) was coupled to the reaction. Acetylated Acs (10 μM) was incubated with CobB (800 nM) with and without *E*. *coli* nicotinamidase (1 μM), and Acs activity was measured for 4 hours. Acs activation rate was 8.5 times higher when nicotinamidase was present (8.95 U/mg in the absence of nicotinamidase and 76.62 U/mg in the presence of nicotinamidase). Thus, Acs total activation time was reduced from 2 hours to 15 minutes when nicotinamide was continuously degraded by nicotinamidase.

**Fig 2:**
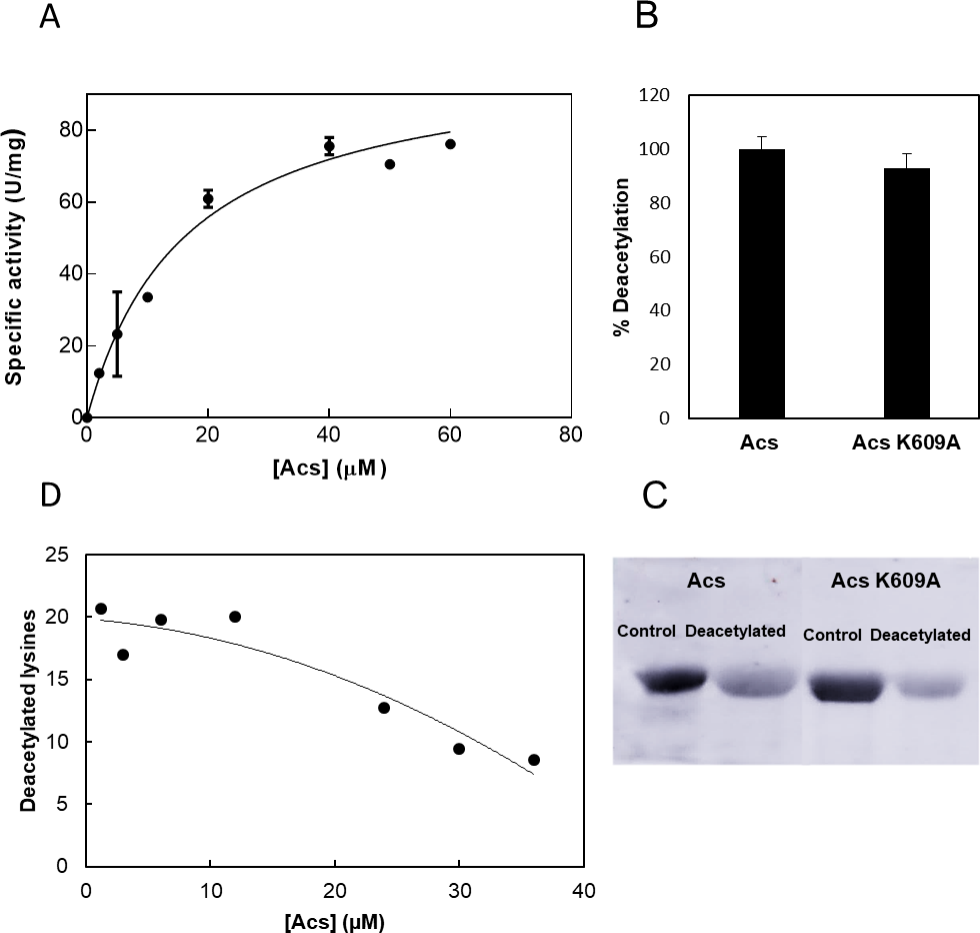
CobB activity on acetylated Acs. **(A)** Substrate saturation curve of CobB at different Acs concentrations. **(B)** Acs and Acs K609A mutant deacetylation (%). **(C)** Western blot with anti-Ac-K of Acs (lanes 1 and 2) and Acs K609A mutant (lanes 3 and 4) before (control) and after CobB deacetylation (deacetylated). **(D)** Number of lysines deacetylated by CobB per Acs molecule at different Acs concentrations.

To study the kinetics of Acs deacetylation by CobB using a continuous assay, the sirtuin reaction was coupled to nicotinamidase and glutamate dehydrogenase activities. It is important to highlight that NAM product inhibition was eliminated with this assay, since nicotinamide is continuously degraded. NADH oxidation was studied at different acetylated Acs concentrations with a constant concentration of NAD^+^ (5 mM, saturated). The CobB concentration was 0.7 μM in the reactions. CobB exhibited hyperbolic Michaelis-Menten kinetics when the acetylated Acs concentration was varied from 0 to 60 μM (Fig 2A). The calculated kinetic parameters are shown in Table 1.

**Table 1:** Kinetic parameters of CobB activity with acetylated Acs as a substrate.

K_m_ (μM)	V_max_ (U/mg)	k_cat_ (s^-1^)	k_cat_/K_m_ (s^-1^ μM^-1^)
16.19 ± 3.23	101 ± 7.66	2404,76	148,53

To know if the results observed corresponded only with K609 deacetylation or with several acetylated lysines, the recombinant protein Acs K609A (lysine K609 was substituted by an alanine residue) was overexpressed and purified from an *E*. *coli* BL21 (DE3) Δ*cobB* strain to achieve a high acetylation level. Acs K609A deacetylation was studied kinetically at a protein concentration of 20 μM with a CobB and NAD^+^ concentrations of 0.7 μM and 5 mM, respectively. The resultant deacetylation activity was compared with the obtained for Acs wt protein. Data are expressed in deacetylation percentage in Fig 2B (100% deacetylation was fixed for Acs wt deacetylation). A western blot assay of the deacetylation reactions was carried out using an anti-acetyl lysine antibody (Fig 2C). The Acs K609A kinetics and western blot assays demonstrated that the kinetics of Acs wt deacetylation by CobB studied are the result of the deacetylation of several Acs acetylated lysines, not only of K609. However, Acs activity was not recovered if K609 was acetylated or mutated, as has been reported for *Salmonella enterica* Acs and other acyl-CoA synthetases [21,31,69,70].

Since enzymatic CobB activity (μmol NAM produced/min), the number of deacetylated lysines per Acs molecule was calculated at each Acs concentration that was assayed (1 NAM produced per an acetylated lysine deacetylated by CobB). The results are shown in Fig 2D. Approximately 20 lysines were deacetylated by CobB when deacetylation showed a linear dependence on Acs concentration (0–12 μM).

### CobB is non-competitively inhibited by nicotinamide

**Fig 3:**
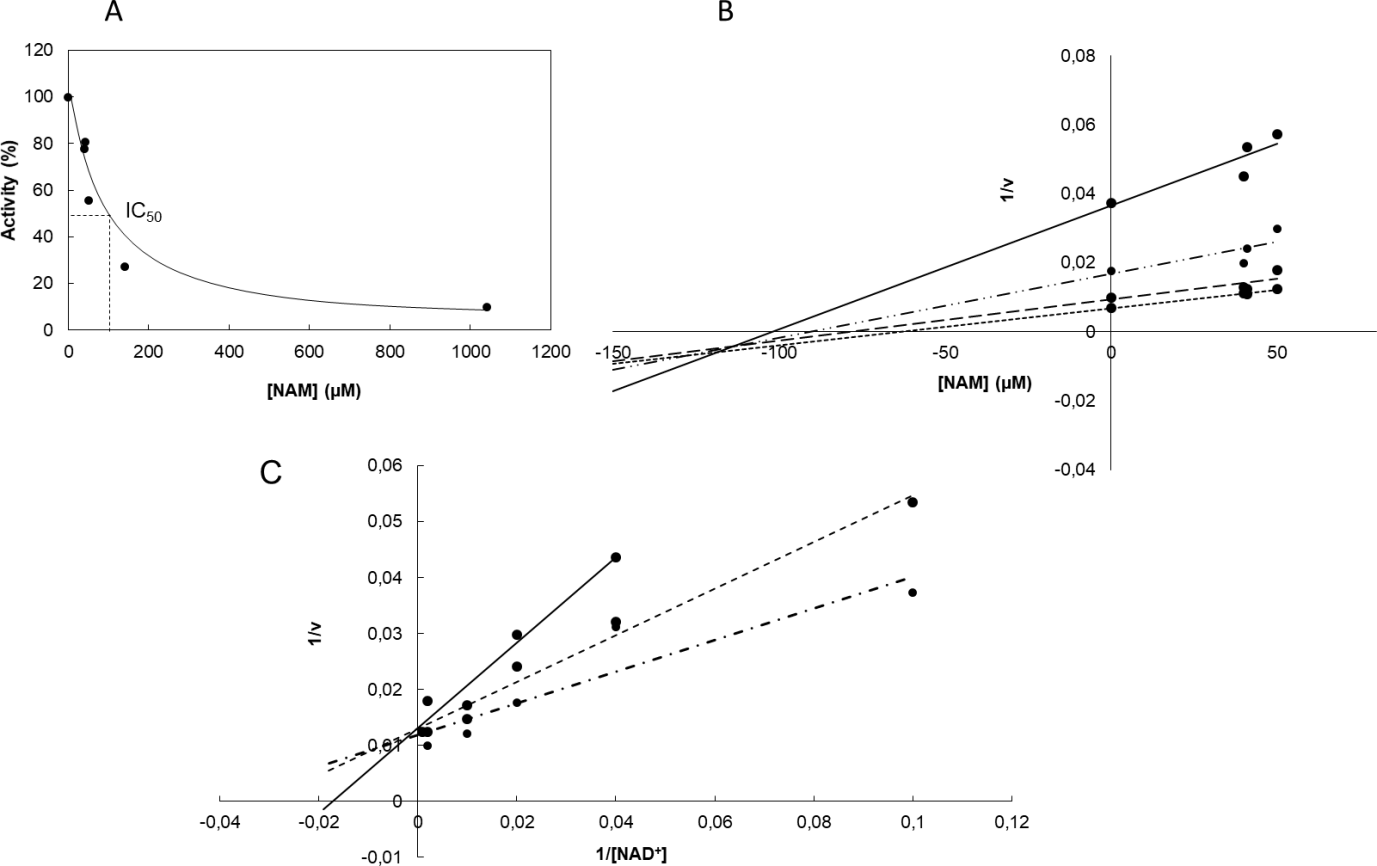
Analysis of CobB inhibition by nicotinamide. **(A)** CobB activity on acetylated Acs at different NAM concentrations. **(B)** Dixon plot ((1/v) vs [NAM]) of CobB deacetylation at different NAD^+^ concentrations: 10 (—), 50 (–∙), 500 (--) and 100 μM (---). **(C)** Double-reciprocal plot (1/v versus 1/ [NAD^+^]) of CobB deacetylation at different NAM concentrations: 0 (-∙∙), 40 (∙∙∙∙∙∙) and 50 (—) μM. Presented data are average of triplicates. Standard deviations were always less than 10%.

Nicotinamide is a sirtuin reaction product and a well-known inhibitor of sirtuins [53–56,67,68]. NAM inhibition of yeast and mammalian sirtuins has been biochemically and kinetically studied [54,55,67,71,72], but to our knowledge, it has not been studied in depth in a bacterial sirtuin. To study CobB nicotinamide inhibition, acetylated Acs was incubated in a 12:1 ratio with CobB. Acs activity was measured after 4 hours of reaction. Nicotinamide concentrations were quantified in a parallel assay employing coupled nicotinamidase and glutamate dehydrogenase reactions. The highest CobB activity (0% inhibition) was assured by coupling the nicotinamidase enzyme. The value was determined by studying CobB activity at 0.5 mM NAD^+^ and different NAM concentrations (Fig 3A). The IC_50_ value was approximately 52 μM. To determine the inhibition constant (k_i_), NAD^+^ concentration was varied from 0 to 1 mM (10, 50, 500 and 1000 μM) at different NAM concentrations and a Dixon plot (1/v vs NAM concentrations at different [NAD^+^]) was made (Fig 3B). The calculated value of k_i_ was approximately 108 μM NAD^+^. To complete the inhibition study, a double-reciprocal plot (1/v versus 1/ [NAD^+^]) at different NAM concentrations was carried out (Fig 3C). These plots suggest that the activity of the CobB sirtuin has a non-competitive inhibition mechanism. This result is in concordance with the transglycosidation mechanism proposed for most examples of sirtuin nicotinamide inhibition [53,56,73].

### Nicotinamide concentrations are high enough to regulate CobB *in vivo*

**Fig 4:**
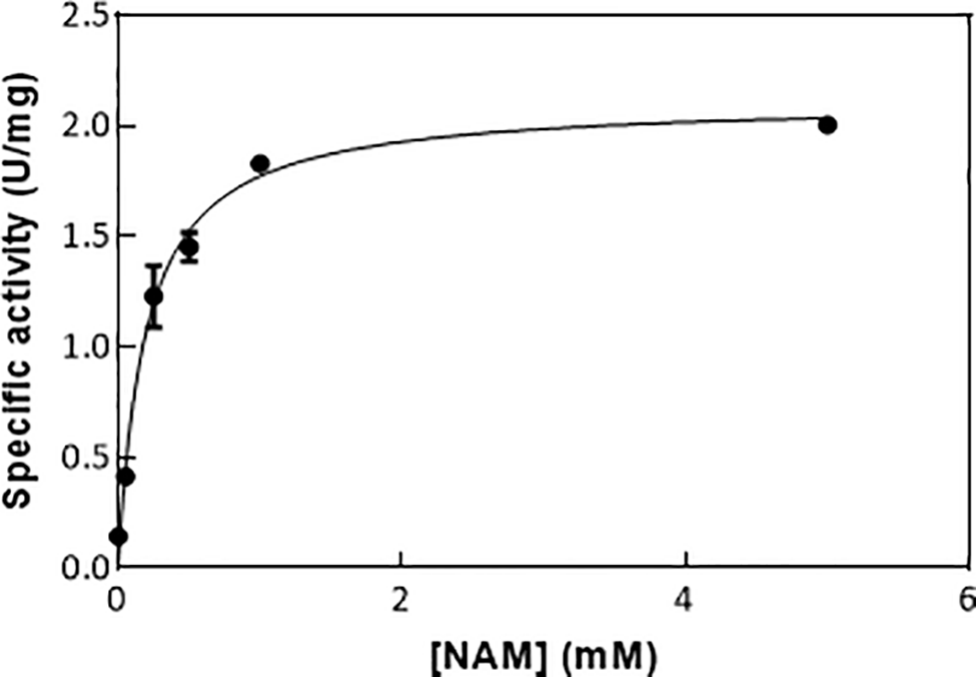
Nicotinamidase kinetics characterization. Substrate saturation curve of nicotinamidase at different nicotinamide concentrations.

Nicotinamide is an *E*. *coli* metabolite in the NAD^+^ salvage pathway, an important route that allows NAD^+^ recycling. NAM is synthetized from NAD^+^ by the protein NMN nucleosidase and is released by nicotinamidase (PncA) to form nicotinamidic acid. In this study, *E*. *coli* PncA has been biochemically characterized. To kinetically characterize PncA, kinetics assays were performed in which the nicotinamide concentration was varied from 0 to 5 mM and the PncA concentration was 0.76 μM. The enzyme showed Michaelis-Menten kinetics with values of 0.175 ± 0.027 mM and 2.05 ± 0.07 U/mg for K_m_ and V_max_, respectively (Fig 4). This Km value is higher than the value reported for nicotinamidase enzymes from other organisms (2–110 μM) [74,75]. PncA inhibition by 3-pyridinecarbonitrile and 3-pyridinecarboxaldehide, a potent irreversible and a reversible competitive inhibitors of *Mycobacterium tuberculosis* nicotinamidase [74], respectively, was evaluated. PncA activity was measured at different nicotinamide concentrations after incubating the enzyme with 1 mM inhibitor for 10 minutes at 37°C. Nicotinamidase activity was undetectable in both assays. This result confirms PncA inhibition by 3-pyridinecarbonitrile and 3-pyridinecarboxaldehide *in vitro*. The relatively high nicotinamidase K_m_ determined in this work could suggest, based on this inhibition study (Fig 3), that *E*. *coli* intracellular nicotinamide concentrations are high enough to regulate CobB activity *in vivo*.

To study *in vivo* CobB regulation by nicotinamide, intracellular nicotinamide concentrations were measured during the exponential and stationary growth phases for both the *E*. *coli* wt strain and a Δ*pncA* mutant growing in minimal and complex media with glucose or glycerol as their carbon sources. The resultant concentrations are summarized in Table 2.

**Table 2:** Intracellular nicotinamide concentrations. Nicotinamide concentrations measured during exponential (X) and stationary (S) growth phases of *E*. *coli* wt and Δ*pncA* mutant strains growing in minimal and complex media with glucose or glycerol as the carbon source.

	wt X	wt S	Δ*pncA* X	Δ*pncA* S
**TB7 + Glucose**	36,51 ± 7,52	38,15 ± 1,66	91,95 ± 4	31,96 ±0,93
**TB7 + Glycerol**	67,89 ± 20,02	28,31 ± 5,03	198,45 ± 24,65	77,65 ±12,87
**MM9 + Glucose**	21,20 ± 2,81		63,82 ± 8,33	33,55 ± 2,3
**MM9 + Glycerol**	33,98 ± 4,26	14,35 ± 2,60	47,03 ± 0,1	46,86 ± 3,06

Nicotinamide concentrations in cells were higher in complex than in minimal medium and higher concentrations were generally found during exponential growth. The Δ*pncA* mutant showed higher nicotinamide concentrations than the wt strain (approximately 2.5 times higher in complex TB7 medium). The maximum concentration was quantified for the *pncA* mutant during the exponential growth phase growing in TB7 complex medium supplemented with glycerol.

### CobB could be regulated *in vivo* by nicotinamide

To study *in vivo* CobB regulation by nicotinamide, physiological characterization of *E*. *coli* wt, Δ*cobB* and Δ*pncA* strains was carried out. Specific growth rates, glucose and glycerol consumption and acetate overflow were analysed for the strains growing in minimal and complex media with glucose or glycerol as carbon source. Specific growth rates in MM9 medium with glucose were lower in the *cobB* mutant than in the wt strain, while significant differences were not observed in the rest of media. The *pncA* mutant also grew slower than *E*. *coli* wt, with a value similar to that observed for Δ*cobB*. The results are shown in Table 3.

**Table 3:** Specific growth rate. Specific growth rate (h^-1^) of *E*. *coli* wt, Δ*cobB* and Δ*pncA* strains growing in minimal and complex media with glucose or glycerol as the carbon source.

Medium	wt	Δ*cobB*	Δ*pncA*
**MM9 + Glucose**	0.72 ± 0.02	0.58 ± 0.02	0.56 ± 0.02
**MM9 + Glycerol**	0.49 ± 0.01	0.48 ± 0.01	0.53 ± 0.02
**TB7 + Glucose**	1.01 ± 0.14	1.07 ± 0.05	0.85 ± 0.03
**TB7 + Glycerol**	0.95 ± 0.13	0.84 ± 0.06	0.77 ± 0.04

**Fig 5:**
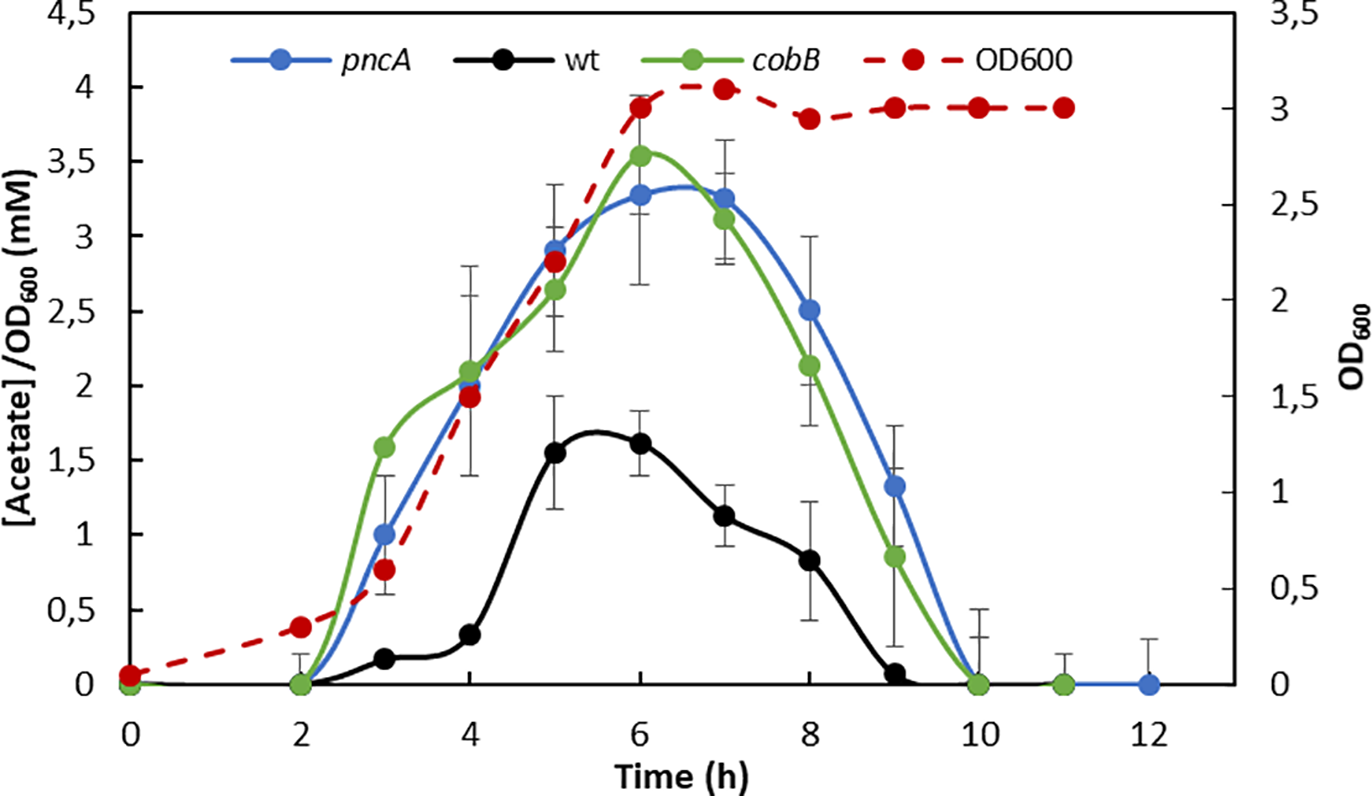
Acetate overflow. Extracellular acetate concentrations (mM) per OD (600 nm) of *E*. *coli* wt (black line), Δ*cobB* (green line) and Δ*pncA* (blue line) strains grown in minimal medium with 20 mM glucose. *E*. *coli* wt growth curve is shown as a discontinuous red line.

Glucose and glycerol consumption were not affected by *cobB* or *pncA* depletion; however, extracellular acetate levels, as a consequence of *E*. *coli* acetate overflow from bacteria growing in MM9 with glucose, were higher in the mutant strains (Fig 5). Observing similar behaviour in the deletion strains suggests total or partial CobB inhibition in the Δ*pncA* strain due to a higher intracellular nicotinamide concentration.

To analyse the acetylation levels in the strains, a western blot of total protein extract, employing an anti-acetyl-lysine antibody, was carried out. The strains were grown in TB7 supplemented and non-supplemented with glucose, and cells were harvested during the mid-exponential (OD_600_ = 1) and stationary phases. The western blot did not show differences in the acetylation of the protein extracts. The similar acetylation patterns in wt and Δ*cobB* strains have been previously detected by other authors [7,15].

**Fig 6:**
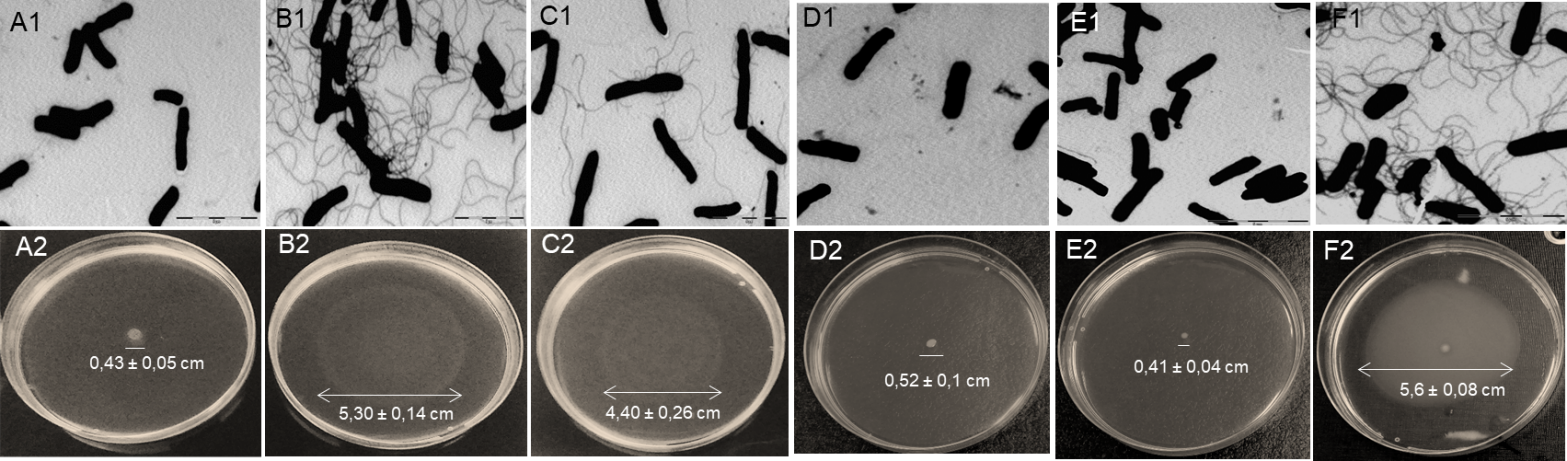
Electron microscopy and migration assays. Electron microscope images of **(A1)** *E*. *coli* wt, **(B1)** Δ*cobB*, **(C1)** Δ*pncA*, **(D1)** Δ*cobB* + *cobB*pBAD24, **(E1)** Δ*pncA*+ *pncA*pBAD24 and **(F1)** Δ*cobB* + *pncA*pBAD24 strains during the exponential growth phase in TB7 medium supplemented with glycerol. Semi-solid agar plates of **(A2)** *E*. *coli* wt, **(B2)** Δ*cobB*, **(C2)** Δ*pncA* strains, **(D2)** Δ*cobB* + *cobB*pBAD24, **(E2)** Δ*pncA*+ *pncA*pBAD24 and **(F2)** Δ*cobB* + *pncA*pBAD24 strains. Migration distances (diameter) and standard deviation from the mean migration of three biological replicates are shown.

To complete the physiological characterization of the *E*. *coli* wt, Δ*cobB* and Δ*pncA* strains, the presence of their flagella was analysed using electron microscopy. CobB regulates the RcsB transcription factor by deacetylating its K154 and the absence of CobB causes an increase in the number of flagella [7,15,40]. We hypothesized that a CobB-inhibited strain would also show an increase in the number of flagella, so electron microscope imaging of the three strains during the exponential growth phase in TB7 with glucose as carbon source was carried out. The results are shown in Fig 6 (A1-C1). The *cobB*-deficient strain had significantly more and longer flagella than the wt strain. *E*. *coli* lacking the nicotinamidase protein also showed a phenotype that was similar to but less pronounced than that of Δ*cobB*. The Δ*pncA* strain had more flagella than the *E*. *coli* wt, but fewer than the Δ*cobB* strain. A migration assay using semi-solid agar plates was employed to quantify cells migration diameter. The assay was carried out on triplicate and the results are shown in Fig 6 (A2-C2). A migration diameter of 0.43 ± 0.05 cm was observed on *E*. *coli* wt plates, while a 5.30 ± 0.14 cm and 4.40 ± 0.26 cm was measured for Δ*cobB* and Δ*pcnA* strains. To verify the effect of *cobB* and *pncA* deletions, the mutant strains Δ*cobB* and Δ*pncA* were transformed with *cobB*pBad24 and *pncA*pBad24, respectively. Cells phenotype was evaluated through electron microscopy and migration assays. The results are shown in Fig 6 (D1, D2, E1, E2). Both of mutant strains (Δ*cobB* and Δ*pncA*) recovered the wt phenotype when were complemented with the expression plasmids. These assays prove that the migration and flagella expression observed in Δ*cobB* and Δ*pncA* are consequence of genes deletion. Finally, an epistasis experiment was carried out to determine if the effect of PncA depends upon CobB. Thus, PncA was overexpressed in the *cob* mutant and phenotype was evaluated with semi-solid agar plates and electron microscopy. Results are shown in Fig 6 (F1 and F2). Cells showed a Δ*cobB* phenotype and no effect was observed with PncA overexpression. This result points out that PncA has not a CobB-independent effect on *Escherichia coli* migration.

## Discussion

Sirtuins are an important family of enzymes that are widely distributed all domains of life. They are involved in many metabolic processes such as glucose or lipid metabolism, DNA repair, transcription regulation and tumour proliferation [76]. The kinetics of sirtuin activity against acetylated peptides have been studied and most of these studies have found a sirtuin selectivity for acetylated peptides substrates [43–48]; however there is no clear evidence of this sequence specificity when native proteins are used as substrates. Thus, one of the biggest challenges in sirtuin research is to determine how specificity is determined in natively folded substrate proteins. In recent years, a few studies have focused on complete and native protein specificity of sirtuins [42,77]. The results suggest that structural protein components are the main determinants of sirtuin specificity. The kinetics study carried out in this work, examining natively folded Acs deacetylation by CobB, is in concordance with this hypothesis. The results have shown a single kinetic phase for several acetylated lysines of Acs, indicating the same binding affinity or specificity for acetylated lysines at different positions (Fig 2). The results of this study and the lack of understanding of the reasons for the discrepancy between the *in vitro* and *in vivo* results suggest that the results of assays carried out using peptides as substrates should be contrasted with new results, which use whole native proteins.

Nicotinamide inhibition of several eukaryotic sirtuins has been studied, and the mechanism of inhibition has been characterized. The mechanism of NAM inhibition is based on nicotinamide intercepting the ADP-ribosyl-enzyme-acetyl-peptide intermediate, resulting in regeneration of the NAD^+^ substrate (transglycosidation) [53]. Several crystal structures of sirtuins with NAM have been determined. In these structures, NAM binds to the peptide-sirtuin reaction intermediate, but the binding location is not clear. In some structures, NAM appears to be bound to the pocket where the NAD^+^ substrate binds (C pocket) despite this binding not being consistent with non-competitive inhibition [67]. In other structures, NAM binds in a pocket other than the NAD^+^-binding pocket [56,72]. The characterization of CobB inhibition suggests that non-competitive nicotinamide inhibition occurs, which would agree with the transglycosidation mechanism. The kinetic parameters (K_i_ and IC_50_) that describe CobB inhibition by nicotinamide have also been determined. The obtained values (IC_50_ = 52 μM and K_i_ = 108 μM) are very similar to those found when nicotinamide inhibits other sirtuins. The K_i_ parameters of hSIRT2 and HST2 are 33.9 and 298 μM [53], respectively, while the IC_50_ values for Sirt1, Sirt2 and Sirt3 have been recently established at 175, 80 and 72 μM [71]. The K_i_ value determined for CobB is also in agreement with the only inhibition study of a bacterial sirtuin (Sir2Af2 from the sulfate-reducing Archaea *Archeaoglobus fulgidus*), which reported a K_i_ value of 26 μM [78]. These inhibition parameters, including the CobB K_i_ calculated in this study, are in the range from 20–300 μM.

Nicotinamidase is a relatively unstudied enzyme in *E*. *coli*. PncA participates in the NAD^+^ salvage pathway in *E*. *coli*, which is a route to NAD^+^ recycling. In this study, nicotinamidase has been kinetically characterized for the first time, and the physiology of the deletion strain Δ*pncA* has been studied. The results showed that a deficiency in nicotinamidase causes several differences between the mutant strain and wt *E*. *coli* and point to a clear similarity between the Δ*pncA* and Δ*cobB* strains. Physiological characterization of wt, Δ*cobB* and Δ*pncA* strains showed that when the mutant strains grew in minimal M9 medium supplemented with glucose, their specific growth rate (Table 3) was much lower than that of the wt strain in the same conditions. The levels of extracellular acetate, a result of *E*. *coli* acetate overflow, were also different in the Δ*cobB* and Δ*pncA* strains than in the wt (Fig 5), being higher in the mutant stains. This fact could be due to low activity by acetylated Acs in these mutants. Acs activity constitutes one of the two routes to assimilate acetate from the extracellular *E*. *coli* medium, where it was excreted to as a consequence of overflow metabolism [79,80]. The absence or inhibition of CobB would cause an increase in the acetylation of Acs that would totally or partially block this acetate pathway [21]. In addition, the flagella and motility in the three studied strains were analysed by electron microscopy and semi-solid agar assays (Fig 6). It has been reported that the transcription factor RcsB is acetylated by acetyl-phosphate and deacetylated by CobB at K154 [15,40,81]. Besides, RcsB K154 acetylation increases the expression of the flagella regulon, provoking an increase in motility and number of flagella. The Δ*cobB* mutant also shows this phenotype when RcsB is acetylated [15]. In this study, the *pncA* deletion strain showed more flagella and motility than the wt strain (a ten-fold increase), but flagella expression and motility were less pronounced than those of Δ*cobB*. This result suggests that in the Δ*pncA* mutant, the transcription factor RcsB could be partially inhibited by lysine acetylation as a consequence of low or no CobB deacetylase activity.

Intracellular nicotinamide concentrations in wt and Δ*pncA* strains have been determined under different conditions. In the wt *E*. *coli* strain, nicotinamide concentrations ranged between 30 and 70 μM during exponential growth phase. Stationary NAM concentrations were always lower (wt MM9 stationary NAM concentrations were not detected). Based on the *in vitro* inhibition of CobB (Fig 3), these concentrations could regulate CobB activity *in vivo*. To our knowledge, this example is the first time that an intracellular bacterial nicotinamide concentration has been determined to date. Previous studies have quantified nicotinamide concentrations in yeast (10–150 μM) [82] and in mammalian tissues (11–400 μM) [83–85]. These concentrations are very similar to those determined in this study in *E*. *coli*. Intracellular nicotinamide concentrations were higher in the Δ*pncA* strain than in wt *E*. *coli* (approximately two times higher). These higher concentrations could justify the physiological behaviour of the *pncA* deletion strain, i.e., conditions in which the CobB protein is totally or partially inhibited. This result suggests that CobB is regulated *in vivo* by nicotinamide concentration. Previous studies have demonstrated that yeast processes regulated by Sir2 are altered by additional copies of the NAD^+^-salvage route genes such as *npt1* (nicotinate phosphoribosyltransferase), *pnc1* (nicotinamidase), *nma1*, and *nma2* (nicotinate mononucleotide adenylyltransfereases 1 and 2) [86], and exogenous nicotinamide [68]. In addition, pnc1 overexpression activate Sir2 *in vivo*, altering *Saccharomyces* longevity [87]. In addition, human breast cancer cell treatment with nicotinamide induces cellular apoptosis *in vivo* through Sirt1 inhibition [88], although a recent study points to the fact that nicotinamide addition to cells can stimulate sirtuins due to rapid *in vivo* conversion of nicotinamide to NAD^+^ [89].

**Fig 7:**
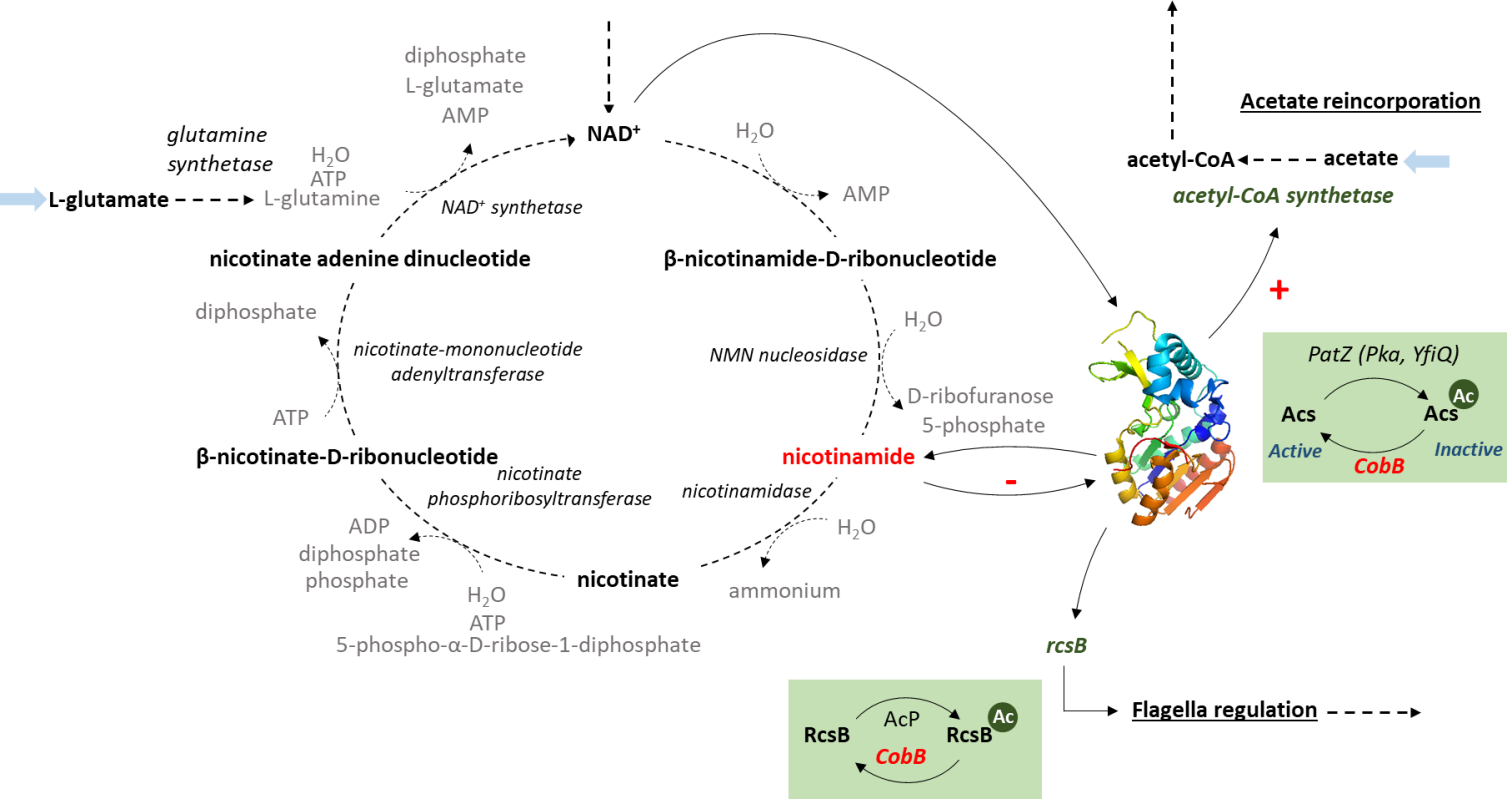
Central role of the nicotinamide metabolite in *E*. *coli* metabolism.

The role of nicotinamide as an *in vivo* inhibitor of CobB activity, as suggested in this study, identifies this metabolite and the nicotinamidase PncA as potential regulators of central *E*. *coli* metabolism due to the global role of CobB (Fig 7). Thus, we have suggested that nicotinamide concentrations could regulate very different aspects of *E*. *coli*, specifically, acetate consumption and flagellum expression. The results of this work open the way for future studies on the regulatory roles of the NAD^+^ salvage route and nicotinamide, and how they affect post-translational acetylation in *E*. *coli*.

## Supporting information

S1 Table Strains, plasmids and primers used in this study. Restriction nuclease sites are in grey. Bold typeface indicates the modified codon during site-directed-mutagenesis.
(PDF)
