# Supplementary material for: Characterization of CobB kinetics and inhibition by nicotinamide
Source: PLoS One. 2017 Dec 18;12(12):e0189689. doi: 10.1371/journal.pone.0189689 (PMC5734772; doi:10.1371/journal.pone.0189689)
Supplement: S1 Table — Restriction nuclease sites are in grey. Bold typeface indicates the modified codon during site-directed-mutagenesis. (PDF) [file pone.0189689.s001.pdf]

S1 Table. Strains, plasmids and primers used in this study. Restriction nuclease sites are in grey. Bold typeface indicates the modified codon during site-directed-mutagenesis.

***E. coli* strain**	**Genotype**	**Source**
BW25113	*lacI* q *rrnB*T14 D*lacZ*WJ16 *hsdR*514 D(*araBAD*)AH33 D(*rhaBAD*)LD78	Keio collection [1]
BW25113 Δ*cobB*	[BW25113] *cobB:kan*	Keio collection [1]
BW25113 Δ*pncA*	[BW25113] *pncA:kan*	Keio collection [1]
BL21 (DE3)	F- ompT gal dcm lon hsdSB(rB- mB-) λ(DE3)	Agilent Technologies
BL21 (DE3) Δ*cobB*	[BL21 (DE3)] *cobB:kan*	[2]
DH10B	F- mcrA Δ(mrr-hsdRMS-mcrBC) Φ80dlacZΔM15 ΔlacX74 endA1 recA1 deoR Δ(ara,leu)7697 araD139galU galK nupG rpsL λ-	Invitrogen
**Plasmid**		
*acs*ASKA	N-terminal, His$_6$-tag overexpression vector, Cam^R. Encodes *acs* wt.	ASKA collection [3]
*cobB*pBAD24-MBP	C-terminal, His$_6$-tag-MBP overexpression vector, Kan^R. Encodes *cobB* wt.	This study
*pncA*pRSETA	N-terminal, His6-tag overexpression vector, Amp^R. Encodes *pncA* wt.	This study
pBAD24cobB	Expression vector Amp^R. Encodes *pncA* wt.	This study
pBAD24pncA	Expression vector Amp^R. Encodes *pncA* wt.	This study
**Primers**	**Sequence**	
*cobB*pBAD24-MBP cloning Fwd	GGTGGTGAATTCATGCTGTCGCGTCGGGGT	
*cobB*pBAD24-MBP cloning Rev	GGTGGTCTCGAGTCAGGCAATGCTTCCCGCT	
*pncA*pRSETA cloning Fwd	GGTGGTCTCGAGATGCCCCCTCGCGCGCCCTG	
*pncA*pRSETA cloning Rev	GGTGGTAAGCTTTTACCCCTGTGTCTCTTCCC	
*acs*K609AASKA mutagenesis Fwd	CTAAAACCCGCTCCGGC**GCA**ATTATGCGCCGTATTC	
*acs*K609AASKA mutagenesis Rev	GAATACGGCGCATAATT**GCG**CCGGAGCGGGTTTTAG	
cobBpBAD24 cloning Fwd	GGTGGTGAATTCATGCTGTCGCGTCGGGGT	
cobBpBAD24 cloning Rev	GGTGGTAAGCTTTCAGGCAATGCTTCCCGCT	
pncApBAD24 cloning Fwd	GGTGGTGAATTCATGCCCCCTCGCGCCCT	
pncApBAD24 cloning Rev	GGTGGTAAGCTTTTACCCCTGTGTCTCTTCCC	

1. Baba T, Ara T, Hasegawa M, Takai Y, Okumura Y, Baba M, et al. Construction of *Escherichia coli* K-12 in-frame, single-gene knockout mutants: the Keio collection. Mol Syst Biol. 2006;2. doi:10.1038/msb4100050

2. Castaño-Cerezo S, Bernal V, Post H, Fuhrer T, Cappadona S, Sánchez-Díaz NC, et al. Protein acetylation affects acetate metabolism, motility and acid stress response in *Escherichia coli*. Mol Syst Biol. 2014;10: 762. doi:10.15252/msb.20145227

3. Kitagawa M, Ara T, Arifuzzaman M, Ioka-Nakamichi T, Inamoto E, Toyonaga H, et al. Complete set of ORF clones of *Escherichia coli* ASKA library (a complete set of *E. coli* K-12 ORF archive): unique resources for biological research. DNA Res. 2005;12: 291–9. doi:10.1093/dnares/dsi012
